# Home Indoor Pollutant Exposures among Inner-City Children With and Without Asthma

**DOI:** 10.1289/ehp.10088

**Published:** 2007-07-27

**Authors:** Gregory B. Diette, Nadia N. Hansel, Timothy J. Buckley, Jean Curtin-Brosnan, Peyton A. Eggleston, Elizabeth C. Matsui, Meredith C. McCormack, D’Ann L. Williams, Patrick N. Breysse

**Affiliations:** 1 Department of Medicine, Johns Hopkins University School of Medicine, Baltimore, Maryland, USA; 2 Department of Epidemiology, Bloomberg School of Public Health, Johns Hopkins University, Baltimore, Maryland, USA; 3 School of Public Health – Environmental Health Sciences, College of Medicine and Public Health, Ohio State University, Columbus, Ohio, USA; 4 Department of Pediatrics, Johns Hopkins University School of Medicine, Baltimore, Maryland, USA; 5 Department of Environmental Health, Bloomberg School of Public Health, Johns Hopkins University, Baltimore, Maryland, USA

**Keywords:** African American, air pollution, allergens, asthma, particulate matter, pediatric, urban

## Abstract

**Background:**

Evidence for environmental causes of asthma is limited, especially among African Americans. To look for systematic differences in early life domestic exposures between inner-city preschool children with and without asthma, we performed a study of home indoor air pollutants and allergens.

**Methods:**

Children 2–6 years of age were enrolled in a cohort study in East Baltimore, Maryland. From the child’s bedroom, air was monitored for 3 days for particulate matter ≤ 2.5 and ≤ 10 μm in aerodynamic diameter (PM_2.5_, PM_10_), nitrogen dioxide, and ozone. Median baseline values were compared for children with (*n* = 150) and without (*n* = 150) asthma. Housing characteristics related to indoor air pollution were assessed by caregiver report and home inspection. In addition, indoor allergen levels were measured in settled dust.

**Results:**

Children were 58% male, 91% African American, and 88% with public health insurance. Housing characteristics related to pollutant exposure and bedroom air pollutant concentrations did not differ significantly between asthmatic and control subjects [median: PM_2.5_, 28.7 vs. 28.5 μg/m^3^; PM_10_, 43.6 vs. 41.4 μg/m^3^; NO_2_, 21.6 vs. 20.9 ppb; O_3_, 1.4 vs. 1.8 ppb; all *p* > 0.05]. Settled dust allergen levels (cat, dust mite, cockroach, dog, and mouse) were also similar in bedrooms of asthmatic and control children.

**Conclusions:**

Exposures to common home indoor pollutants and allergens are similar for inner-city preschool children with and without asthma. Although these exposures may exacerbate existing asthma, this study does not support a causative role of these factors for risk of developing childhood asthma.

Asthma is common among children in the United States, especially those who are racial/ethnic minorities living in inner cities (Centers for Disease Control and Prevention 2004). Although definitive causes of asthma remain to be discovered, substantial evidence points to environmental exposures, which may in turn interact with individual genetic susceptibility—a phenomenon often called gene–environment interaction. Some evidence points to the heritability of asthma, because it occurs more commonly in children whose parents have asthma and in affected twins (Laitinen et al. 1998). Because not all cases of asthma arise in people with affected first-degree relatives, however, it leaves open the possibility that asthma results from high exposure to environmental factors. Evidence to support asthma causation through high exposure alone can come from studies that compare the environment of people with and without asthma.

Environmental studies of inner-city children are especially important for understanding asthma disparities among African Americans. Asthma prevalence is 39% higher among African Americans than whites, and African Americans are more likely than whites to live in urban areas (86% vs. 70% of the respective populations), areas which are at high risk for air pollution (American Lung Association 2005). Indeed, 65% of African Americans live in counties that failed to meet at least one of the U.S. Environmental Protection Agency’s (EPA) outdoor air quality standards (American Lung Association 2005). The home indoor environment is especially relevant for studying inner-city childhood asthma, because some pollutants, such as ambient particulate matter (PM), penetrate from the outside, and some are generated and remain indoors, such as particles and gases from smoking, heating, cooking, and cleaning (Abt et al. 2000; Howard-Reed et al. 2000; Long et al. 2000; National Research Council and Committee on Research Priorities for Airborne Particulate Matter 2004; Rea et al. 2001; Vette et al. 2001). Remarkably, a previously published study from the Center for Childhood Asthma in the Urban Environment, conducted in older children from inner-city Baltimore (90% African American), has already shown that children are exposed to concentrations of indoor PM that are three times the concentrations found in outdoor air (Breysse et al. 2005). Such concentrations would frequently exceed the outdoor limits set by the U.S. EPA (Breysse et al. 2005). Furthermore, the indoor environment may be especially critical to study because Americans, including preschool children, spend the vast majority of time indoors.

Studies are urgently needed to determine the causes of the asthma epidemic, and in response to this need, there has been support for research from the U.S. federal government to uncover the role of environmental exposures in the etiology and prevention of prevalent disorders, such as asthma, in children (National Institute of Environmental Health Sciences 2003). To address this issue, the Johns Hopkins Center for Childhood Asthma in the Urban Environment conducted a study to determine whether indoor home environmental pollutants are greater in homes of preschool children with asthma compared with homes of children without asthma.

## Methods

### Study population

We recruited children 2–6 years of age who resided in urban area of Baltimore, Maryland, defined by 9 contiguous zip codes. Children with and without asthma were identified using a two-stage screening method. All children who were patients of health systems that provide care to most East Baltimore residents were identified from billing records. If the child had had a health care encounter for asthma [*International Classification of Diseases, 9th Revision* (ICD-9) code 493.x (World Health Organization 1975)] in the previous 12 months, he or she was considered a potential asthma subject. Asthma status was confirmed, for the purposes of this study, if the primary caregiver also reported that the child met both of the following criteria: *a*) doctor-diagnosed asthma and *b*) symptoms of asthma and/or medication use for asthma in the previous 6 months. Control subjects were those selected from the same health systems using billing records, who had not had a health care encounter for asthma and for whom the caregiver reported that the child had never had doctor-diagnosed asthma.

### Recruitment procedures

Recruitment for this study occurred between September 2001 and December 2003. Recruitment continued until the planned sample of 300 subjects was achieved. A letter was sent to the parents of children identified as potential subjects. The letter described the study aims and general requirements, and a postcard was enclosed to return if the parent did not wish to hear more about the study. Two weeks after the initial letter, study staff attempted to contact the parent by phone. By phone, the study was described in greater detail, and a screening survey was used to determine eligibility. If the subject was eligible, a home visit was scheduled for obtaining informed consent, administration of a detailed health and environmental survey, home inspection, and collection of environmental samples. An appointment was made for the clinical assessment of the child, including allergy skin testing. A detailed protocol for finding correct addresses and phone numbers was employed, and multiple mailings and calls were attempted. The study was approved by the Johns Hopkins School of Medicine Institutional Review Board. Informed consent was obtained from all participants before the study. For successful completion of the study, caregivers received $30 and the child received a toy valued at $10. In addition, all subjects received detailed information about environmental measurements in their homes, and subjects with asthma received information about disease severity and allergy skin test results. Study design and implementation were reviewed and guided by a community advisory board.

### Home inspection

After obtaining informed consent from the primary caregiver and before any sampling, a trained environmental health technician inspected the home using an inspection checklist (Mitchell et al. 1997) to document housing characteristics and condition of the house. In addition to general housing conditions, the home inspection also focused on an assessment of the kitchen, TV/living room, and the child’s bedroom.

### Air sampling

We conducted air sampling over a 72-hr period in the sleeping room of the child. PM_10_ (PM with an aerodynamic size ≤ 10 μm) and PM_2.5_ (PM with an aerodynamic size ≤ 2.5 μm) samples were collected using 4-L/min MSP impactors (St. Paul, MN) loaded with 37-mm, 2.0-μm pore size, PALL Teflo PTFE membrane filters with polypropylene support rings (Pall Corp., Ann Arbor, MI). Inlet flows were checked at the beginning and end of each sampling period using primary standards (BIOS DryCal; Bios International Corp., Butler, NJ). Samples were collected using pumps plugged into house electrical service to assure 72 hr of operation. Ozone and nitrogen dioxide were sampled passively. All sampling heads and passive badges were attached to the outside of a sampling frame that was placed in a convenient location in the child’s bedroom. In most cases the sampling frame was placed on the dresser or a nightstand. In some cases, when there was no available elevated surface, the sample frame was placed on a portable stand constructed out of PVC (polyvinyl chloride) pipe.

We conducted PM gravimetric analysis using a Mettler T5 microbalance (Mettler, Columbus, OH). Before analysis, filters were placed in petri dishes and stored for 24 hr in a weighing room equipped with temperature and humidity controls. Time-resolved PM was also assessed using a portable direct-reading nephelometer with data-logging capability (MIE pDR1000s; ThermoElectron, Franklin, MA). The instrument incorporates a pulsed, high-output, near-infrared light-emitting diode source (880 nm). The intensity of the light scattered over the forward angle inside the inlet by the particles passing through the sensing chamber is linearly proportional to the airborne PM concentration. The instrument’s optical configuration produces response to particles in the size range of 0.1–10 μm, although empirical evidence suggests that there is a differential response such that particles in the size range of 0.3–2 μm are more efficiently detected relative to the size fraction from 2 to 10 μm (Howard-Reed et al. 2000; Liu et al. 2002; Quintana et al. 2000). The instrument was operated in the passive sampling mode and has a measurement range of 1.0–400,000 μg/m^3^.

We monitored O_3_ using a small (2 × 3 cm) and lightweight (7-g) passive sampler (Ogawa badge) (Koutrakis et al. 1993). The method is based on O_3_’s reaction with a nitrite-coated filter to yield nitrate, which is then quantified by ion chromatography. Samplers and coated filters were purchased from Ogawa, Inc. (Pompano Beach, FL). Air is effectively sampled at a rate of 22.8 cm^3^/min. The limit of detection (LOD) was calculated based on the analysis of field blanks. The median LOD was 3.1 ppb for a 72-hr sample.

We measured NO_2_ with the same Ogawa passive monitors used for O_3_ monitoring, but with a different configuration and loaded with filters coated with triethanolamine (TEA) (Palmes et al. 1976). In the presence of a color reagent, NO_2_ and TEA form a highly colored azo dye that is measured spectrophotometrically at 540 nm. The median LOD calculated from the analysis of field blanks was 6.8 ppb for a 72-hr sample.

### Allergen collection and analysis

Household dust samples were collected and assayed for the allergens of cat (Fel d 1), dog (Can f 1), cockroach (Bla g 1), dust mite (Der p 1 and Der f 1), and mouse (Mus m 1) in the laboratory of P.E. (Eggleston et al. 1998; Perry et al. 2003). Dust samples were collected on an unwoven fabric collector inserted into the nozzle of a typical portable vacuum. Samples were collected from the child’s bedroom using established methods (Wood et al. 2001). The bedroom sample was collected by vacuuming a 1-m^2^ area near and underneath the bed for 2 min combined with a 2-min sample from the mattress and bedding. After sampling, the fabric collector was removed from the vacuum and sealed in a plastic bag.

An aqueous extract of 100 mg of the sieved dust (sieve size, 300 μm) specimen was prepared in 2 mL borate-buffered saline. The extracts were stored at −30°C until they were assayed for Fel d 1, Can f 1, Bla g 1, Der p 1, Der f 1, and Mus m 1 using antibody-based ELISA (Chapman et al. 1988; Ohman et al. 1994; Pollart et al. 1991; Wood et al. 1988). The results were expressed in micrograms per gram of dust or (for Bla g 1) in units per gram of dust. The limits of detection of the assay were 50 ng/g for Fel d 1, Can f 1, Der p 1, and Der f 1; 2.2 ng/g for Mus m 1; and 1 U/g for Bla g 1.

### Clinical assessment

We used an interviewer-administered questionnaire to assess household demographics, housing characteristics, potential sources of indoor pollutants, indoor environmental exposures (including allergens), child’s respiratory symptoms, and medication use. To assess environmental exposures over the child’s lifetime, we asked care-givers whether specific exposures relevant to indoor pollution were present in the child’s home over several time periods: during pregnancy, child’s age 0–12 months, child’s age 1–2 years, child’s age 2 years until 1 month ago, and at present (within the previous month).

We determined atopic status by skin prick testing (Multi-Test II; Lincoln Diagnostics, Decatur, IL) for 13 aeroallergens: American and German cockroach, dust mite mix, cat, dog, mouse, rat, 3 pollens, and 3 molds (Hollister-Stier Laboratories, Spokane, WA; and Greer Laboratories, Lenoir, NC). A positive skin test was defined as a wheal size of at least 2 mm greater than the negative control. Because our institutional review board does not allow allergy skin testing in children without asthma, the control children did not undergo allergy skin testing.

### Statistical analysis

After ascertaining whether or not distributions were normally or near normally distributed, we compared continuous variables using Student’s two-tailed *t*-test or the Kruskal-Wallis test, as appropriate. We determined differences for categorical variables by Pearson’s chi-square or Fisher’s exact test, as appropriate. To determine whether season of collection confounded results, we also compared pollutant concentrations between asthma and control homes separately by season. All analyses were performed with StataSE version 8.0 (StataCorp., College Station, TX). A two-tailed *p*-value < 0.05 was used to detect statistically significant differences.

## Results

A total of 300 subjects with (*n* = 150) and without (*n* = 150) asthma were enrolled. Children with and without asthma were quite similar by sociodemographic factors (Table 1), including a mean age of 4.4 years in each group. Only sex was significantly different, with more boys in the asthma group, as expected based on the greater prevalence of asthma among males in childhood. Among children with asthma, 69% were atopic and 92% had wheezing in the preceding 12 months, including 41% who wheezed when they did not have a cold or the flu. Of control children, only 1% had wheeze when they did not have a cold or the flu, suggesting an extremely low rate of potential misclassification by disease status.

### Housing characteristics

On average, children lived in their current homes for almost 3 years (3.0 years for asthmatic children, 2.6 years for controls). Approximately one-third of children had lived in the same home their whole life (36.7% of children with asthma, 30.7% of controls). The predominant housing type was a row house. Some houses had evidence of deterioration, with broken plaster (21%) and cracks or holes in the walls and/or doors (22%) being most common.

### Housing characteristics related to indoor pollutant exposure

There were no significant differences in potential sources of pollutants in homes of asthmatic compared with control children as measured by either caregiver report or home inspection (Table 2). Most homes demonstrated evidence of indoor pollutant sources, including gas stove use and signs of indoor tobacco smoking. More than two-thirds of homes were within 25 feet of the curb (71% of asthma homes and 69% of non-asthma homes; *p* = 0.95), and one-quarter of homes were on arterial streets (27% of asthma homes and 23% of non-asthma homes; *p* = 0.73).

Comparing children with asthma to controls, we found little difference in any of the potential sources of pollutants reported by caregivers at all intervals of the child’s earlier life (pregnancy, 0–12 months, 1–2 years, 2 years to 1 month ago, present) (Table 3).

### Indoor pollutant concentrations

Measured indoor pollutant concentrations (PM_2.5_, PM_10_, NO_2_, O_3_) were similar in bedrooms of asthmatic and control children (Table 4). Even when stratified by season of collection, there still were no significant differences in pollutant concentrations by asthma and control status, with one exception. In samples collected in the summer, NO_2_ was higher in asthmatic than control homes (*p* < 0.05). Median daily and peak time–resolved PM values using the nephelometer were also similar in asthmatic and control homes. The percentage of homes with PM_2.5_ concentrations above the U.S. EPA’s (1997) 24-hr average National Ambient Air Quality Standard (NAAQS) of 65 μg/m^3^ was 14.1% in homes of children with asthma and 16.8% in homes of children without asthma (*p* = 0.54). Pollutant concentrations in the homes of asthmatic and control children who lived in the same home for their whole life were not different compared with those who had moved at least once (data not shown).

### Indoor allergen exposure

Indoor allergen exposures, measured by caregiver report, home inspection, or dust allergen levels, were similar in homes of asthmatic and control children (Table 5). Mice and cockroaches were commonly reported, but furry pets (cats and dogs) were uncommon.

## Discussion

Our study indicates that, in a population of predominantly African-American, inner-city preschool children, exposures to common home indoor pollutants are similar in those with and without asthma. Based on caregiver report, we found no evidence for differences in potential sources of these exposures across the lifespan of the children beginning *in utero*. Although these exposures may exacerbate existing asthma, this study does not suggest that high indoor pollutant exposure alone is a risk for developing childhood asthma. Indoor exposure could still play a critical role in the development of asthma among genetically susceptible individuals through gene environment interaction. Several candidate genes have been found to interact with exposure to air pollution in both *in vivo* human and mouse models (Koppelman 2006; Yang et al. 2007). It remains to be seen whether or not a population of genetically susceptible individuals would still develop asthma if the *in utero* and early childhood environment had lower indoor pollutant concentrations. There are also many other potential indoor exposures, including fungi, endotoxin, and other agents, which may still have a role in asthma pathogenesis.

Although much of the focus of childhood asthma research has been on the development of allergic responses to common indoor allergens, there are good reasons to suspect a role for etiologic pathways that involve nonallergic mechanisms. Evidence from some studies has shown an association of asthma incidence with outdoor air pollutants ( O_3_, PM, and NO_2_) (Rios et al. 2004). Although most people spend most of their time indoors, there is some evidence that indoor-generated particles may be more bioactive than ambient particles (Long et al. 2001). In one previous European study, active smoking, passive smoking, and using coal for cooking/heating were associated with incident asthma, suggesting that indoor pollution may play a causal role in asthma development (Zejda and Kowalska 2003). It should also be noted that burning of biomass fuels for cooking and heating in the developing world has been linked to respiratory symptoms and chronic respiratory diseases in children (Bruce et al. 2000). Thus, the investigation of indoor pollutants as factors causing asthma has appeared to be promising.

Recently, another study of asthmatic children (school-age rather than preschool-age) children in Baltimore demonstrated exposure to high concentrations of indoor air pollutants. Our present study confirms that high levels of exposure to indoor pollutants in inner-city children are also present in a population of younger children. However, the present study is unique in that there were nonasthmatic controls and there were no significant differences in the indoor air pollutant concentrations (PM_2.5_, PM_10_, O_3_, NO_2_), nor in potential sources of indoor air pollution, at present or in early life, between homes of asthmatic and control children. Thus, although certain indoor pollutants can clearly increase asthma morbidity (Gold 2000; Zanobetti et al. 2000), our results are inconsistent with the hypothesis that exposure to these specific indoor air pollutants is sufficient for the development of asthma.

Although there is previous evidence of an association of indoor housing characteristics with asthma and asthma symptoms (Baker and Henderson 1999; Dennekamp et al. 2001; Pilotto et al. 1997) our results are consistent with those of an English epidemiologic study (The Indoor Pollutants, Endotoxin, Allergens, Damp and Asthma in Manchester; IPEADAM) of children 4–17 years of age, which showed little difference in indoor pollutants (respirable suspended particles, tobacco-specific particles, volatile organic compounds, and NO_2_) in homes of asthmatic and nonasthmatic children (Tavernier et al. 2006). Similar to our study as well, the IPEADAM study showed no significant differences in housing characteristics such as pets, heating, cooking fuel, and reported smoking habits. Likewise, another interview-based study in England had previously failed to show an association of home environmental factors with asthma, including gas cooking, pets, and heating type (Butland et al. 1997).

A strength of our study is the comprehensive evaluation of both indoor pollutant and allergen levels in a highly relevant inner-city population of primarily African-American children. But several limitations must also be considered. This study is cross-sectional, which limits causal inference. However, our results were similar when analyzing the pollutant concentrations in homes of children who lived in the same home their whole life, which suggests that the study results are not explained by caregivers of asthmatic children having actively sought an environment with lower exposure. Recall bias, which can affect cross-sectional studies, seems not to be at issue in the present study, because caregiver-reported exposures in the child’s early life were similar between asthmatic and control children. Because asthma diagnosis does not, unfortunately, rely on a gold standard, studies of asthma have the potential for misclassification of asthma. However, participant report of physician-diagnosed asthma has been the main criterion of asthma in many epidemiologic studies of children (McConnell et al. 2006; Merchant et al. 2005), and the validity of this approach, assessed by the repeatability response, is good (Ehrlich et al. 1995). Furthermore, almost none of the control children reported symptoms of wheeze, suggesting an extremely low rate of misclassification by disease status. A particular strength of the present study is the broad range of potential risk factors that were measured, including multiple pollutants and allergens. There are few studies reporting indoor air pollution exposures for asthmatic children in inner-city environments, and only rarely have studies reported on the combined exposures to allergens and indoor air pollutants in asthmatic children. Finally, although our indoor monitoring was limited to 3 consecutive days, studies have shown that classification of exposure based on indoor measurements are relatively stable (i.e., the variability between homes is much greater than within homes). This stability occurs because indoor source activities (e.g., smoking, cooking, housekeeping) patterns tend to be consistent from day to day (Janssen et al. 1997; Lioy et al. 1990; Wallace et al. 1994). We also observed consistency of indoor exposure over time in Baltimore city homes. In an asthma intervention study conducted by Eggleston et al. (2005), average indoor PM concentrations in 50 control-arm homes varied by < 4% across a 12-month period (measurements at baseline, 6 months, and 12 months). There can still be significant variability in indoor PM concentrations due to variability in outdoor levels, but within a region the indoor exposure classification remains relatively constant because ambient PM is homogeneously distributed and the stable indoor concentrations are superimposed on top of the ambient contribution (Ott et al. 2000).

In summary, our study showed that the indoor environments of children with and without asthma are remarkably similar. It is still possible, of course, that the studied indoor pollutants in genetically susceptible individuals may be sufficient to initiate the disease. The results of this study should not dissuade clinicians and policy makers from continuing to work toward improvement in certain aspects of the home environment for the sake of children with existing asthma (Eggleston et al. 2005; Morgan et al. 2004). Strong evidence supports environmental tobacco smoke as a key contributor to asthma morbidity, as well as outdoor pollutants including PM, NO_2_, and O_3_. Families should also continue to avoid allergens such as dust mite allergens and furry pet and pest allergens when the child shows evidence of allergic sensitization.

## Figures and Tables

**Table 1:** Child and caregiver characteristics (%).

Characteristic	Asthma (*n* = 150)	Control (*n* = 150)	*p*-Value
Child			
Age [mean (range)]	4.4 (2–6)	4.4 (2–6)	0.82
Male sex	58.0	42.7	0.01
Race			0.76
Black	91.2	88.7	
White	4.7	6.7	
Other	4.1	4.0	
Allergy skin test (positive)			
German or American cockroach	43		
Mouse	17		
Rat	25		
Cat	30		
Dog	9		
Dust mite	25		
*Aspergillus fumigatus* 15			
*Helminthosporium interseminatum*	8		
*Penicillim notatum*	8		
*Alternaria tenius*	19		
Grass pollen	29		
Easter oak	14		
Ragweed pollen	11		
Caregiver/family			
Primary caregiver			0.72
Birth mother	87.1	84.7	
Grandmother	4.8	8.0	
Birth father	2.7	2.7	
Other	5.4	4.6	
Employment			0.36
Work full-time	34.5	34.2	
Unemployed	20.3	23.5	
Homemaker	18.2	18.1	
Work part-time	12.8	12.1	
Household income (annual)			0.23
< $25,000	41.6	45.3	
$25,000–$50,000	10.8	12.2	
> $50,000	2.0	5.4	
Unknown/refused	45.6	37.1	
Health insurance			0.62
Private	11.5	8.8	
Public	86.5	89.2	
Self-pay	1.4	2.0	

**Table 2:** Housing characteristics related to sources of indoor air by caregiver report and home inspection (%).

Characteristic	Asthma	Control	*p*-Value
Range/stove			
Gas			
Caregiver report	83.0	86.6	0.39
Home inspection	87.3	88.3	0.81
Electric			
Caregiver report	20.4	17.8	0.51
Home inspection	12.7	11.7	0.81
Heating fuel (caregiver report)			
Gas	72.0	73.3	0.57
Electric	17.4	12.0	0.26
Oil	8.7	11.3	0.45
Other	3.4	4.0	0.76
Heating			
Radiators			
Caregiver report	30.7	28.2	0.55
Home inspection	30.8	26.2	0.39
Forced air			
Caregiver report	53.7	57.7	0.07
Home inspection	57.3	57.9	0.92
Forced air, no filter			
Caregiver report	7.3	4.7	0.21
Home inspection	0.7	0.7	0.99
Other			
Caregiver report	0.10	0	0.32
Home inspection	11.2	15.2	0.32
Smoking in home			
Caregiver report			
Any smoker in home	55.7	60.0	0.45
Mother	32.7	30.7	0.71
Father	20.0	22.0	0.84
Other	27.3	29.3	0.70
Home inspection			
Ashtray/cigarette butts in home	23.4	29.7	0.23
Smell of smoke in home	19.4	23.4	0.42

**Table 3:** Housing characteristics related to sources of indoor air pollution during child’s earlier life (%).

	Pregnancy		0–12 months		1–2 years		2 years–1 month ago	
	Asthma	Control	Asthma	Control	Asthma	Control	Asthma	Control
Range/stove								
Gas	82	84	84	85	81	84	82	85
Electric	20	18	20	18	22	21	23	20
Heating fuel								
Gas	76	74	76	74	73	73	73	73
Electric	13	13	14	12	16	13	16	13
Oil	7	9	7	11	10	11	9	12
Other	3	4	2	4	2	4	2	4
Heating								
Radiators	25	23	27	23	29	23	31	28
Forced air	57	65	56	67	55	65*	55	59
Forced air, no filter	8	3	8	3	7	3	8	4
Other	1	1	1	1	1	1	1	1
Smoking	43	48	49	57	54	59	54	63

* *p* < 0.05 for comparison of asthma vs. control.

**Table 4:** Air pollutant concentrations in bedroom at present [median (interquartile range)].

	Sampling device	Asthma	Control	*p*-Value
Bedroom air [72-hr average (μg/m^3^)]				
PM_2.5_	MSP Impactor with filter	28.7 (18–51)	28.5 (17–50)	0.99
PM_10_	MSP Impactor with filter	43.7 (29–70)	41.1 (27–68)	0.35
Time-resolved PM	Nephelometer			
Peak		20 (10–40)	20 (10–40)	0.93
NO_2_ (ppb)	Passive sampling badge	21.6 (14–34)	20.9 (14–31)	0.84
O_3_ (ppb)	Passive sampling badge	1.4 (0.9–3.4)	1.8 (0.9–4.1)	0.56

**Table 5:** Allergen and allergen-related exposures at present.

Allergen	Asthma	Control	*p*-Value
Dog			
Caregiver report (%)	16.0	15.3	0.87
Inspection (%)	16.8	16.6	0.96
Bedroom settled dust^a^ (ng/g)	115	86	0.36
Cat			
Caregiver report (%)	24.7	23.3	0.59
Inspection (%)	21.0	24.8	0.44
Bedroom settled dust^a^ (ng/g)	448	534	0.59
Cockroach			
Caregiver report (%)	45.3	46.7	0.82
Inspection			
Live (kitchen) (%)	16.2	17.4	0.79
Dead (kitchen) (%)	9.9	12.5	0.48
Bedroom settled dust^a^ (U/g)	3.2	3.8	0.78
Mouse			
Caregiver report (%)	65.3	62.7	0.63
Inspection			
Rodent (%)	1.4	5.5	0.06
Mouse droppings (kitchen) (%)	31.0	39.3	0.14
Bedroom settled dust^a^ (ng/g)	2,562	2,978	0.65
Dust mite			
Bedroom settled dust^a^ Der p 1 (ng/g)	Below detection	Below detection	—
Bedroom settled dust^a^ Der f 1 (ng/g)	28.5	40.5	0.78

a Settled dust measures are median values.
